# Transgenerational transmission of behavioral phenotypes produced by exposure of male mice to saccharin and nicotine

**DOI:** 10.1038/s41598-020-68883-6

**Published:** 2020-07-20

**Authors:** Deirdre M. McCarthy, Sarah E. Lowe, Thomas J. Morgan, Elisa N. Cannon, Joseph Biederman, Thomas J. Spencer, Pradeep G. Bhide

**Affiliations:** 1 Biomedical Sciences and Center for Brain Repair, College of Medicine, Florida State University, 1115, West Call Street, Tallahassee, FL 32306 USA; 2 Pediatric Psychopharmacology, Department of Psychiatry, Massachusetts General Hospital, Harvard Medical School, Boston, MA 02114 USA; 3 Present Address: School of Physician Assistant Practice, College of Medicine, Florida State University, 1115, West Call Street, Tallahassee, FL 32306 USA

**Keywords:** Cognitive neuroscience, Molecular neuroscience

## Abstract

The use of non-nutritive sweeteners such as saccharin is widely prevalent. Although saccharin is considered safe for human consumption, it produces behavioral changes in experimental animals. We report that saccharin’s behavioral effects are much more pervasive than currently recognized. In a mouse model, saccharin exposure produced motor impulsivity not only in the saccharin-exposed males but also in their offspring. In addition, the offspring showed locomotor hyperactivity and working memory deficit not observed in fathers. Spermatazoal DNA was hypermethylated in the saccharin-exposed fathers, especially at dopamine receptor promoter regions, suggesting that epigenetic modification of germ cell DNA may mediate transgenerational transmission of behavioral phenotypes. Dopamine’s role in hyperactivity was further highlighted by the finding that the stimulant drug methylphenidate mitigated the hyperactivity. Nicotine is another substance that is widely used. Its use via smokeless tobacco products, some of which contain saccharin, is on the rise contributing to concerns about adverse outcomes of co-exposure to saccharin and nicotine. We found that co-exposure of male mice to saccharin and nicotine produced significant behavioral impairment in their offspring. Thus, our data point to potential adverse neurobehavioral consequences of exposure to saccharin alone or saccharin and nicotine for the exposed individuals and their descendants.

## Introduction

Nearly 40% of adults in the United States consume low-calorie sweeteners such as saccharin^1^. Although saccharin is not metabolized in the body and although it is regarded as safe for human consumption, it activates brain’s reward circuitry^2,3^, suggesting that saccharin may influence neurobehavioral phenotypes. Nicotine is another substance that is widely used and its use via traditional cigarettes, smokeless tobacco products, and electronic cigarettes is on the rise, especially among the youth and young adults^4–7^. Given the wide prevalence of nicotine and saccharin use, the risk of co-exposure to saccharin and nicotine remains very high. In fact, some smokeless tobacco products contain saccharin at levels nearly 25-fold higher than its levels in food products^8^ and expose the user to both saccharin and nicotine. Approximately 3% of adults were current users of smokeless tobacco products in 2016^9^. Despite these noteworthy statistics, the mental health implications of exposure to saccharin alone or co-exposure to saccharin and nicotine have not received the attention they may deserve.

Preclinical models of oral nicotine exposure employ saccharin as a sweetener to mask the bitter taste of nicotine^10–22^. Thus, co-exposure to nicotine and saccharin occurs in a number of preclinical models. Many of the preclinical studies were focused on the effects of developmental exposure of the offspring to saccharin or nicotine^10–13,18–23^. As a result, female animals were exposed to saccharin alone or nicotine and saccharin. These studies did not report significant effects of exposure to saccharin on the mother or the offspring. However, the co-exposure produced significant behavioral, neurochemical and neuroanatomical changes in the offspring, which were attributed to the effects of developmental nicotine exposure^10–13,18–23^.

The majority of the research on the adverse effects of environmental exposures has focused on women rather than men because exposure of women of childbearing age or during pregnancy can increase the risk of harmful consequences not only for the exposed female but also for her offspring. In other words, when females are exposed to environmental insults, two generations can be exposed to significant risks simultaneously.

Until recently, the risk associated with exposure of males to adverse environmental factors was considered to be limited to the exposed individuals, with little direct impact on the descendants. However, compelling evidence is accumulating that exposure of males to environmental insults such as stress, addictive substances, endocrine disruptors and nutritional deprivation produces adverse impacts not only on the exposed male but also on multiple generations of descendants, even when the female parent was not exposed to the environmental insults^13,26–32^.

Saccharin exposure of males is reported to produce impulsivity^24,25^ and changes in brain’s reward circuitry^2,3^. To the best of our knowledge, the effects of co-exposures to saccharin and nicotine in males have not been reported. Moreover, neither the effects of exposure to saccharin alone nor the effects of co-exposure to saccharin and nicotine on offspring derived from the exposed males have been reported. The risk of co-exposure to saccharin and nicotine is significantly greater for men than for women because more men smoke cigarettes and use other tobacco products than women^6,7^. Therefore, understanding whether exposure of the male parent to saccharin alone or co-exposure to saccharin and nicotine produces adverse effects on the offspring assumes considerable significance.

With this background, we exposed male mice to saccharin alone or saccharin plus nicotine and discovered shared as well as unique behavioral phenotypes in the exposed generation as well as in their offspring. The transgenerational transmission (from the exposed generation to their descendants) of the behavioral phenotypes was associated with epigenetic modification of DNA of the spermatozoa, especially at promoter regions of dopamine receptor genes.

## Results

### Water consumption and body weights

**Figure 1:**
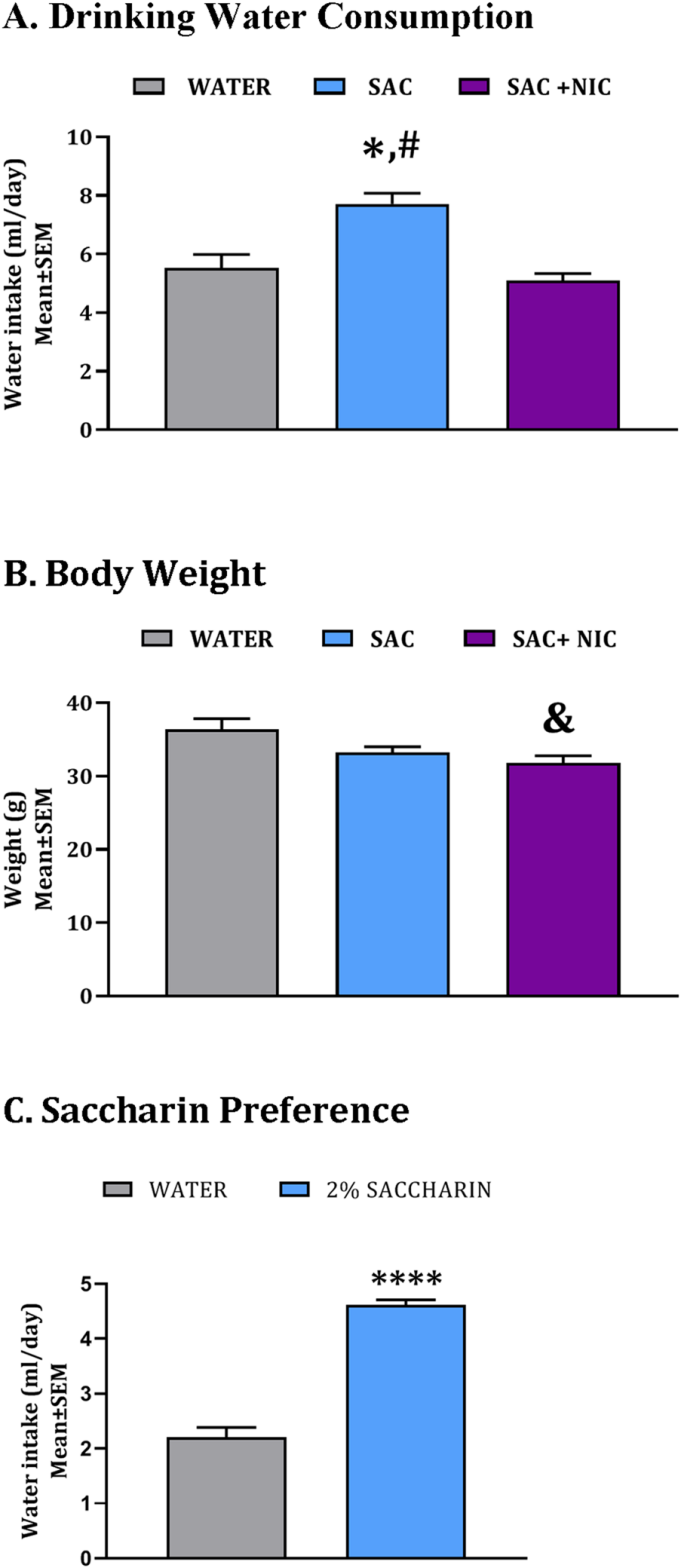
Water consumption, body weight and saccharin preference in F0 mice. Average daily water consumption (ml/day) was calculated over the 12-week exposure period in three groups of male mice each group receiving ad libitum supply of plain drinking water (WATER), drinking water containing 2% saccharin (SAC) or drinking water containing 2% saccharin plus 200 µg/ml nicotine (SAC + NIC) (**A**). Body weight (g) at the end of a 12-week drinking water exposure period was measured (**B**). In a two-bottle choice paradigm, average daily intake (ml/day) of plain drinking water or drinking water containing 2% saccharin was calculated over a 3-week period (**C**). Symbols to indicate statistical significance (*p* < 0.05) in Bonferroni post hoc comparisons *WATER vs. SAC; ^#^SAC vs. SAC + NIC; ^&^WATER vs. SAC + NIC. ****p < 0.0001; Student’s *t*-test. n = 4–6 per treatment group.

We exposed 8-week old male mice (C57BL/6) to drinking water containing 2% saccharin, 2% saccharin plus 200 µg/ml nicotine, or plain drinking water. The three groups will be referred to as saccharin (SAC), saccharin + nicotine (SAC + NIC) and plain drinking water (WATER) groups. The drinking water exposures continued for 12 weeks, during which time we measured water consumption daily. We found significant main effects of the type of drinking water on water consumption (one-way ANOVA: F_(2,36)_ = 7.522, *p* < 0.01). Post hoc comparisons showed that the SAC group consumed significantly greater volume of water compared to the WATER or SAC + NIC groups (WATER vs. SAC, *t* = 3.208, df = 36 *p* < 0.01; SAC vs. SAC + NIC, *t* = 3.492, df = 36, *p* < 0.01; Fig. 1A). The drinking water consumption by the WATER and the SAC + NIC groups was comparable to that by mice drinking water that contained nicotine only (200 µl/ml) in our previous study^26^. Analysis of body weights at the end of the 12-week exposure period showed significant main effect of the drinking water exposure (one-way ANOVA; F_(2,11)_ = 4.529, *p* < 0.05). Post hoc comparisons showed significant decrease in body weight in the SAC + NIC group compared to the WATER group (*t* = 2.953, df = 11, *p* < 0.05; Fig. 1B).

To determine if the mice showed a preference for drinking water containing 2% saccharin, in a separate experiment, mice were given a choice between a bottle containing plain drinking water and another containing water with 2% saccharin. The mice showed a significant preference for the saccharin containing water (two-tailed *t*-test: mean (ml/day) ± SEM: water: 2.21 ± 0.18; saccharin: 4.62 ± 0.09, *t* = 12.17, df = 14, *p* < 0.0001; Fig. 1C) suggesting that exposure to a single water bottle containing 2% saccharin in our studies did not “force” the mice to consume a potentially unpalatable or aversive substance.

### Behavioral analyses in male mice in the WATER, SAC and SAC + NIC groups (F0 generation)

**Figure 2:**
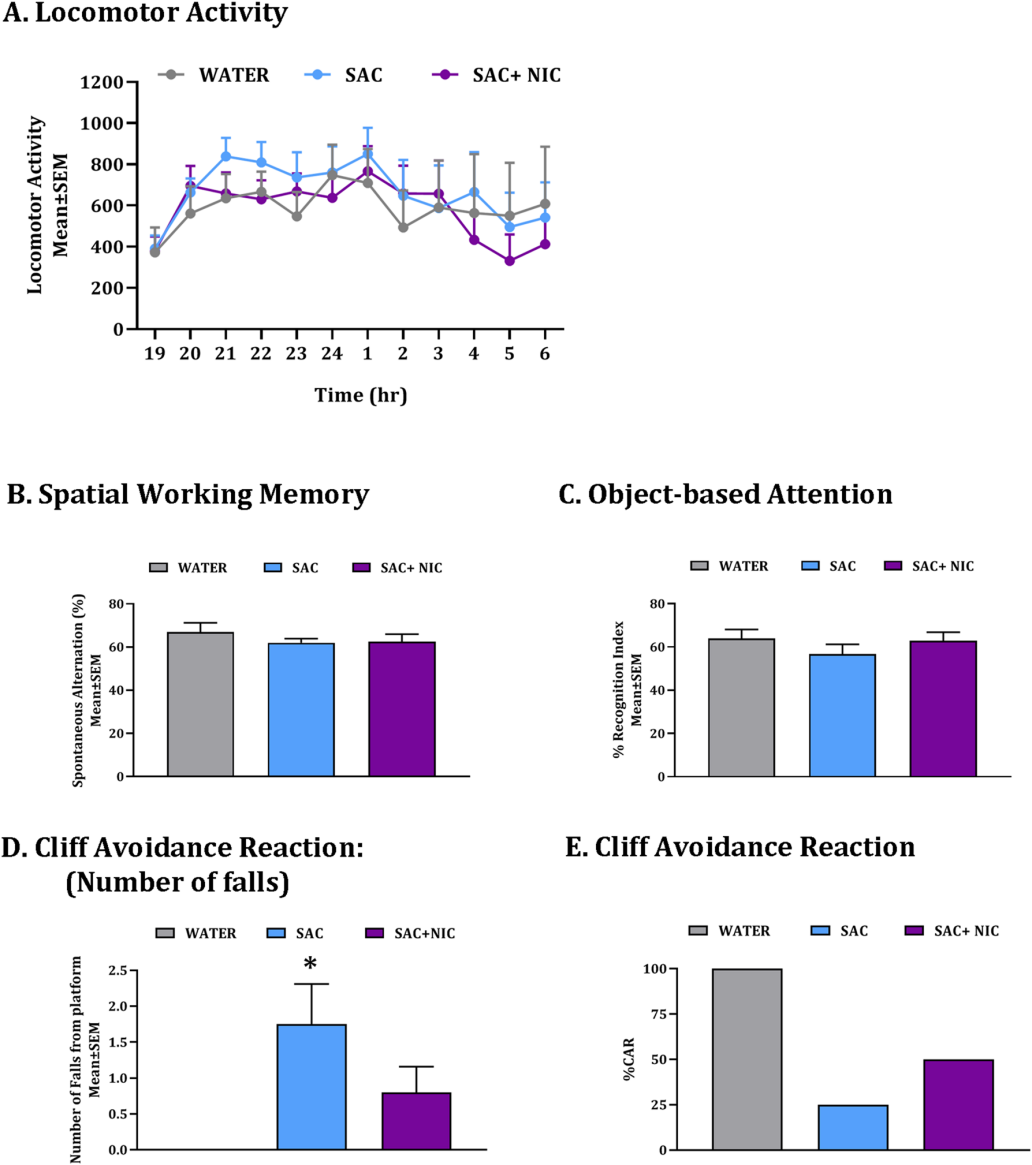
Behavioral phenotypes in F0 mice. Spontaneous locomotor activity was analyzed over a 12-h lights off period (19:00–7:00) in a testing chamber equipped with an activity monitoring system (**A**) in male mice receiving ad libitum supply of plain drinking water (WATER), drinking water containing 2% saccharin (SAC) or drinking water containing 2% saccharin plus 200 µg/ml nicotine (SAC + NIC). Spatial working memory was assayed in a Y-maze (**B**) and attention was analyzed using an object-based attention test (**C**). Motor-impulsivity was assayed using the cliff avoidance reaction (CAR) based on the number of falls from the platform (**D**) and percentage intact CAR (E). *p < 0.05; one-sample *t*-test; SAC significantly different from zero; n = 8–12 per treatment group.

Since exposure to nicotine produces significant changes in locomotor activity and cognitive function^33–38^, we analyzed spontaneous locomotor activity, spatial working memory, attention and motor impulsivity in the three groups of mice. The spontaneous locomotor activity was analyzed over a 12-h lights-off period^10,11,13^ when the mice are naturally more active. There was no significant effect of the type of drinking water (mixed-effects analysis: F_(22,38)_ = 0.0025, *p* > 0.05) but there was a significant effect of time (F_(11,307)_ = 3.714, *p* < 0.0001) reflecting natural variation in activity over the 12 h period. The water × time interaction was not significant (F_(22,307)_ = 0.5569, *p* > 0.05; Fig. 2A). Analysis of spontaneous alternation index, which is the unit of measure of spatial working memory in a Y-maze^10,12,26^, did not show significant effects of the drinking water treatment (one-way ANOVA: F_(2,27)_ = 0.6093, *p* > 0.05, Fig. 2B). Analysis of the recognition index, the unit of measure of attention in the object-based attention test^10,12,26^, also did not show significant effects of the drinking water treatment (one-way ANOVA: F_(2,27)_ = 0.8257, *p* > 0.05; Fig. 2C). Thus, 12-weeks of exposure to saccharin alone or co-exposure to saccharin and nicotine did not produce significant changes in spontaneous locomotor activity, spatial working memory or object-based attention in the male mice. The spatial working memory and object based attention data for the WATER group (Fig. 2B,C) were reported in an earlier study^26^, as the WATER F0 mice for the earlier and present study were raised simultaneously.

Next, we examined cliff avoidance reaction, which is a behavioral measure of the natural tendency of rodents to avoid falls from an elevated platform. Impaired cliff avoidance reaction is a measure of motor-impulsivity^10^, which is correlated with impaired pre-pulse inhibition, a measure of behavioral dis-inhibition or impaired control/suppression in mouse models^39^. We analyzed the number of falls from the elevated platform during a 60 min test period and calculated the cliff avoidance reaction index. The mice in the WATER group had zero falls, indicating an intact cliff avoidance reaction. We used a one-sample *t*-test to compare the mean number of falls in the SAC and SAC + NIC groups to zero (which is the expected mean number of falls based on the WATER group data). The mice in both the experimental groups fell off the platform, and the number of falls in the SAC group was significantly different from zero (t = 3.130, df = 7, *p* < 0.05; Fig. 2D). However, the number of falls in the SAC + NIC group was not significantly different from zero, although the *p* value approached 0.05 (t = 2.228, df = 9, *p* = 0.053; Fig. 2D). Thus, the number of falls in mice in the SAC group but not the SAC + NIC group were significantly different from the WATER group. All of the mice in the WATER group had intact cliff avoidance reaction (i.e. zero falls) whereas only 25% of the mice in the SAC group and only 50% in the SAC + NIC group had intact cliff avoidance reaction (Fig. 2E). Thus, among the behavioral phenotypes analyzed, only the cliff avoidance reaction showed significant effects of 12-weeks of exposure to saccharin alone and co-exposure to saccharin and nicotine in the F0 male mice.

### Behavioral analyses in offspring derived from the F0 mice (F1 generation)

**Figure 3:**
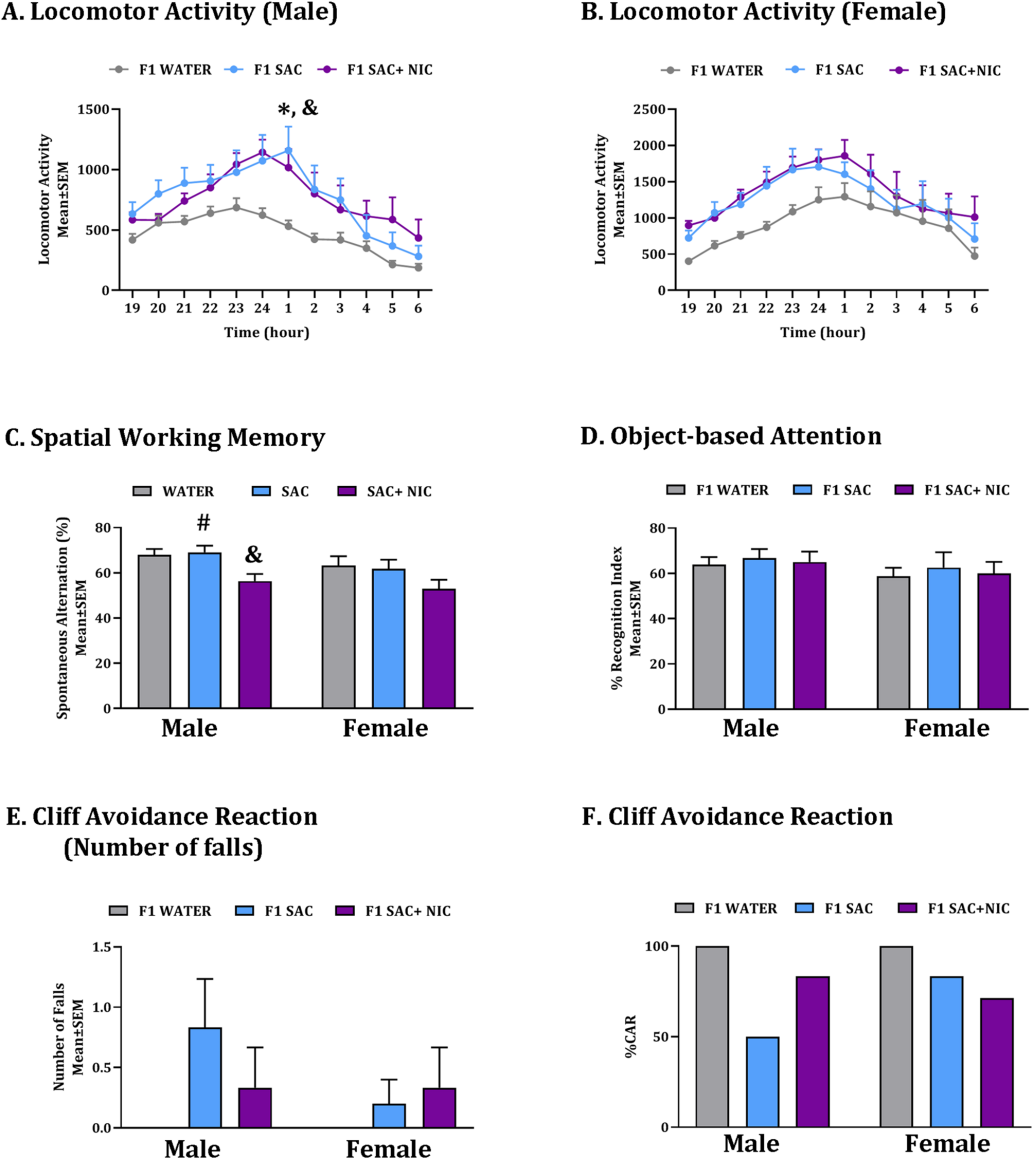
Behavioral phenotypes in F1 mice. The analysis was performed on male and female F1 mice derived from male F0 founder mice receiving ad libitum supply of plain drinking water (WATER), drinking water containing 2% saccharin (SAC) or drinking water containing 2% saccharin plus 200 µg/ml nicotine (SAC + NIC). Spontaneous locomotor activity was analyzed over a 12-h lights off period [19:00–7:00; (**A**, **B**)]. Spatial working memory was assayed using a Y-maze (**C**) and attention using an object-based attention test (**D**). Motor-impulsivity was assayed using the cliff avoidance reaction (CAR) based on the number of falls from the platform (**D**) and percentage intact CAR (**E**). Symbols to indicate statistical significance (*p* < 0.05) in Bonferroni post hoc comparisons: *WATER vs. SAC; ^#^SAC vs. SAC + NIC; ^&^WATER vs. SAC + NIC; n = 8–12 per treatment group.

Male mice from each of the F0 WATER, SAC and SAC + NIC groups were bred with drug naïve female mice following 8- or 12-weeks of exposures to produce the F1 generation. The 8 weeks of exposure is longer than the estimated duration of spermatogenesis in mice, which is about 5 weeks^40–42^. Therefore, the 8-week exposure would cover the entire spermatogenesis cycle. When the F1 offspring were approximately 60 days of age, we performed analysis of spontaneous locomotor activity, spatial working memory, object-based attention and motor-impulsivity, because our studies in other mouse models showed that exposure to nicotine produced significant changes in these phenotypes in the F0 as well as the F1 generation^10–12,14,26^. We used male and female F1 offspring derived from 3–4 litters (each litter produced by a different F0 founder) from each of the 3 paternal treatment groups at each of the 2 durations of paternal exposures. There was no significant difference in any of the behavioral phenotypes among F1 male or female offspring derived from SAC, SAC + NIC or WATER groups following 8-weeks of exposures (data not shown). However, we found significant differences among the 3 groups when the paternal exposures lasted for 12 weeks (Fig. 3), and these data are described below.

There was no significant main effect of 12-weeks of paternal treatment on litter metrics or developmental milestones of the F1 offspring (Table 1). Spontaneous locomotor activity during the 12-h dark phase of the light–dark cycle was analyzed at hourly intervals separately for male and female F1 mice. In male F1 mice, we found a significant main effect of paternal treatment (repeated measures 2-way ANOVA: F_(2,30)_ = 4.280, *p* < 0.05) and time (F_(11,330)_ = 13.88, *p* < 0.0001) but the paternal treatment × time interaction was not significant (F_(22,330)_ = 1.111, *p* > 0.05). Post hoc comparisons revealed that F1 male mice from the paternal SAC and SAC + NIC groups had significant increases in spontaneous locomotor activity compared to the F1 mice from the paternal WATER group (WATER vs. SAC: t = 2.584, df = 30, *p* < 0.05; WATER vs. SAC + NIC: t = 2.542, df = 30, *p* < 0.05; Fig. 3A). Mice in the SAC group and SAC + NIC group were not significantly different from each other (t = 0.0419, df = 30, *p* > 0.05; Fig. 3A). In contrast, in the female F1 mice, there was no significant main effect of paternal treatment (F_(2,25)_ = 1.857, *p* > 0.05) or treatment × time interaction (F_(22,275)_ = 0.6590, *p* > 0.05). However, there was a significant main effect of time (F_(11,275)_ = 17.27, *p* < 0.0001), which was also the case for F1 male mice.

**Table 1:** Litter metrics and developmental milestones in F1 offspring (mean ± SEM).

	Water	Saccharin	Nicotine + saccharin
Litter size on P0			
# Pups	6.75 ± 1.25	7.14 ± 1.09	7.29 ± 0.58
Body weight (g)			
P0	1.35 ± 0.11	1.32 ± 0.06	1.22 ± 0.06
P4	2.45 ± 0.20	2.71 ± 0.19	2.55 ± 0.24
P7	4.17 ± 0.86	4.65 ± 0.30	4.65 ± 0.20
P14	6.64 ± 0.20	7.31 ± 0.47	6.77 ± 0.46
P21	10.56 ± 0.77	10.60 ± 0.53	11.72 ± 1.36
Developmental milestones (# days)			
External ear detachment	3.50 ± 0.40	4.00 ± 0.00	4.00 ± 0.00
Appearance of fur	5.50 ± 0.41	6.33 ± 0.36	6.50 ± 0.16
Eye opening	15.33 ± 0.33	14.71 ± 0.57	14.75 ± 0.34

The size of the litter at birth, body weights of the offspring at different postnatal days (P) until weaning and pre-weaning developmental milestones were analyzed for mice derived from each of the three groups of F0 founders.

*P* postnatal day.

Analysis of the Y-maze data showed a significant main effect of paternal treatment on spontaneous alternations, an index of spatial working memory (two-way ANOVA; F_(2,67)_ = 6.715, *p* < 0.01). However, there was no significant main effect of sex (F_(1,67)_ = 3.128, *p* > 0.05) or significant paternal treatment × sex interaction (F_(2,67)_ = 0.1502, *p* > 0.05). Post hoc comparisons revealed that F1 male mice derived from the paternal SAC + NIC group had a significant reduction in spontaneous alternations compared to the F1 male mice from the paternal WATER (t = 2.490, df = 67 *p* < 0.05) and paternal SAC (t = 2.577, df = 67 *p* < 0.05; Fig. 3C) groups. None of the other post hoc comparisons were significant (Male: WATER vs. SAC: t = 0.0255, df = 67, *p* > 0.05, Female: WATER vs. SAC: t = 0.292, df = 67, *p* > 0.05, WATER vs. SAC + NIC: t = 2.008, df = 67, *p* > 0.05; SAC vs. SAC + NIC: t = 1.778, df = 67, *p* > 0.05).

Analysis of the object-based attention data showed no significant main effects of paternal treatment (two-way ANOVA; F_(2,37)_ = 0.2730, *p* > 0.05), sex (F_(1,37)_ = 1.606, *p* > 0.05) or paternal treatment × sex interaction (F_(2,37)_ = 0.9949, *p* > 0.05) on recognition index, the unit of measure of attention in this assay.

Motor-impulsivity was analyzed using the cliff avoidance reaction test. Similar to F0 male mice from the WATER group (Fig. 2D), neither F1 male nor F1 female mice from the paternal WATER group fell off the platform (zero falls, the expected mean) indicating an intact cliff avoidance reaction (Fig. 3D). The number of falls in F1 mice derived from the paternal SAC or SAC + NIC groups were not significantly different from zero (one-sample *t*-test; t = 2.171, df = 11, *p* = 0.053; SAC + NIC: ; t = 1.806, df = 12, *p* > 0.05; Fig. 3D). All of the F1 mice from the paternal WATER group had intact cliff avoidance reaction, whereas only 66.7% of the F1 mice in the SAC group (male 50%, female 83.3%) and 77.4% of the F1 mice in the SAC + NIC group (male 83.3%, female 71.4%) showed intact cliff avoidance reaction (Fig. 3E).

### Effect of methylphenidate on spontaneous locomotor activity in male F1 mice from the paternally SAC group

**Figure 4:**
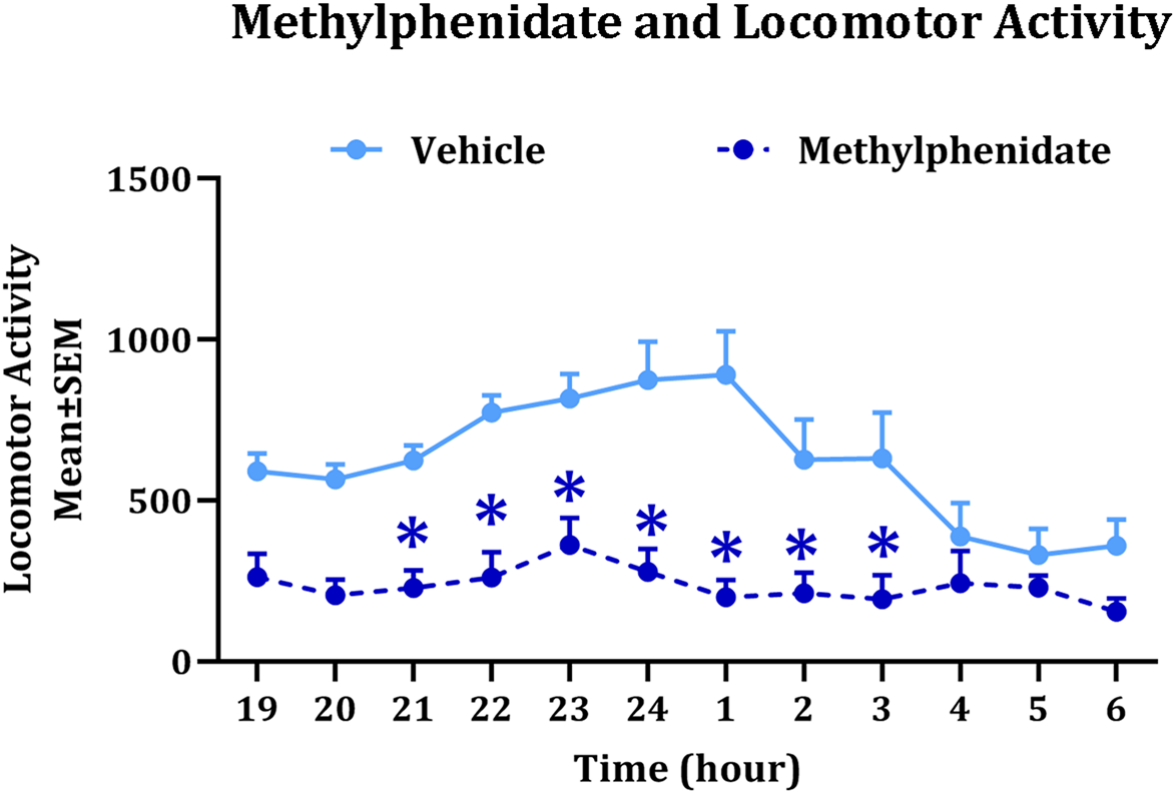
Effect of methylphenidate on spontaneous locomotor activity. The analysis was performed on male F1 mice derived from male F0 founder mice receiving ad libitum supply of plain drinking water (WATER) or drinking water containing 2% saccharin (SAC). The locomotor activity was analyzed over a 12-h lights off period (19:00–7:00) following delivery of vehicle (VEH; sterile saline) or methylphenidate (MPH; 0.75 mg/kg) by oral gavage 15 min prior to commencement of testing. * *p* < 0.05; Bonferroni post hoc comparisons. n = 5–7 per treatment group.

Since F1 male mice derived from the SAC and SAC + NIC groups showed significant increases in spontaneous locomotor activity compared to the F1 male mice from the WATER group, and since locomotor hyperactivity associated with paternal nicotine exposure is consistent with changes in dopamine receptor signaling in the brain^26^, we examined the effects of the classic stimulant methylphenidate (0.75 mg/kg; oral gavage) or saline (vehicle; oral gavage) on spontaneous locomotor activity in the F1 mice from the paternal SAC group. Methylphenidate alleviates hyperactivity in mouse models by increasing dopamine content in the brain. The 0.75 mg/kg dose for a mouse is equivalent to the dose used therapeutically in humans^43^. We found a significant main effect of drug treatment (repeated measures 2-way ANOVA: F_(1,10)_ = 18.57, *p* < 0.01), time (F_(11,110)_ = 5.642, *p* < 0.0001) and drug treatment × time interaction (F_(11,110)_ = 3.501, *p* < 0.001). Post hoc comparisons revealed that methylphenidate produced significant decreases in locomotor activity at each hourly timepoint between 21.00 h and 4:00 h during the lights off period (*p* < 0.05) compared to saline (Fig. 4). Thus, the increase in locomotor activity in the F1 SAC group is likely associated with changes in dopamine neurotransmission.

### DNA methylation in F0 spermatozoa

**Figure 5:**
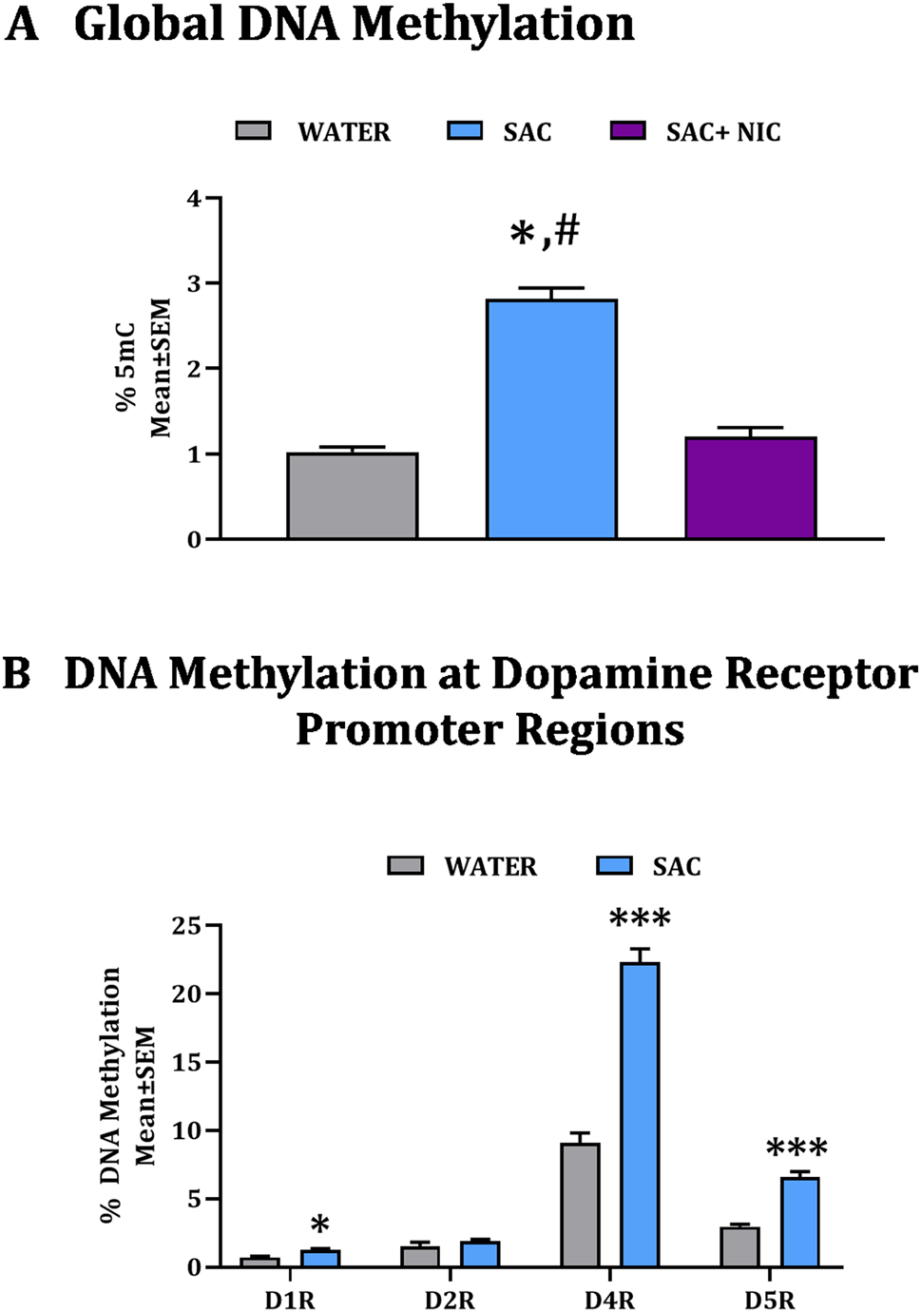
DNA methylation in spermatozoa of F0 mice. The analysis was performed on male mice receiving ad libitum supply of plain drinking water (WATER), drinking water containing 2% saccharin (SAC) or drinking water containing 2% saccharin plus 200 µg/ml nicotine (SAC + NIC). Total DNA methylation (**A**) and DNA methylation at promoter regions of dopamine D1, D2, D4 and D5 receptors (**B**) was analyzed. Statistical significance (*p* < 0.05) in Bonferroni post hoc comparisons of total DNA methylation (**A**): *WATER vs. SAC; ^#^SAC vs. SAC + NIC. Statistical significance in Student’s *t*-test for DNA methylation at each dopamine receptor (**B**): *p < 0.05, ***p < 0.001; n = 3–4 per treatment group.

We analyzed total DNA methylation (percent 5-methyl cytosine content) in spermatozoa isolated from F0 male mice from the WATER, SAC and SAC + NIC groups. We found a significant main effect of experimental treatment (one-way ANOVA: F_(2,9)_ = 99.94, *p* < 0.0001; Fig. 5A) on this measurement. Post hoc comparisons showed significant increase in total DNA methylation in the SAC group compared to WATER and SAC + NIC groups (WATER vs. SAC t = 12.84, df = 9 and SAC vs. SAC + NIC: t = 11.54, df = 9, *p* < 0.0001 for both; Fig. 5A) but the difference between WATER and SAC + NIC groups was not significant (t = 1.304, df = 9, *p* > 0.05; Fig. 5A). The total DNA methylation data for the WATER group (Fig. 5A) were reported in an earlier study^26^, as the WATER F0 mice for the earlier and present study were raised simultaneously.

Our previous work demonstrated that nicotine exposure produced significant changes in DNA methylation at promoter regions of dopamine receptor genes in the spermatozoa^26^. The effects of saccharin exposure on this parameter were not known. We found that DNA methylation was significantly increased at promoter regions of dopamine D1 (t = 4.011, df = 4, *p* < 0.05), D4 (t = 10.97, df = 4, *p* < 0.001) and D5 (t = 8.786, df = 5, *p* < 0.001) receptor genes in the SAC group compared to the WATER group (Fig. 5B). The promoter region of the dopamine D2 receptor gene did not show significant changes (t = 1.049, df = 6, *p* > 0.05; Fig. 5B), and the D3 receptor promoter region was not examined. DNA methylation at promoter regions of dopamine receptor genes in the spermatozoa derived from the SAC + NIC group was not examined.

## Discussion

Our data show that 12-weeks of exposure to saccharin alone or saccharin plus nicotine produces behavioral phenotypes in male mice and in male and female F1 offspring derived from the male mice. Heritability of the phenotypes was influenced by the sex of the F1 offspring as well as the type of exposure of the F0 mice (SAC or SAC + NIC). For example, only male F1 mice derived from the SAC and SAC + NIC F0 groups showed significant locomotor hyperactivity. Working memory deficits also were observed only in the F1 male mice, but only if they were derived from F0 SAC + NIC mice. Another interesting finding was that the behavioral phenotypes in the F0 founders did not predict the phenotypes inherited by the F1 mice. For example, neither spontaneous locomotor hyperactivity nor working memory deficit was present in either the SAC or SAC + NIC F0 mice, but those phenotypes were present in their F1 descendants. Saccharin exposure produced significant increases in total DNA methylation and DNA methylation at promoter regions of dopamine D1, D4 and D5 receptor genes in the spermatozoa of the F0 mice. The locomotor hyperactivity in the F1 male mice derived from the F0 SAC group was ameliorated by methylphenidate, suggesting involvement of dopamine signaling mechanisms in this transgenerational phenotype. The duration of the paternal exposure played a role in the heritability of the behavioral phenotypes in that 12- but not 8-weeks of exposure produced heritable phenotypes. Thus, paternal exposure to saccharin alone or co-exposure to saccharin and nicotine produce heritable phenotypes in male and female mice in the F1 generation.

The concentration of nicotine in drinking water used here (200 µg/ml) produces plasma cotinine level of approximately 80 ng/ml^14,26^, which is consistent with plasma cotinine levels in other rodent models of nicotine exposure^14,26^ as well as in humans smoking 20–26 cigarettes daily^44,45^. On the other hand, the concentration of saccharin (2%) in drinking water used here exceeds the US Food and Drug Administration’s recommendation of 5 mg/kg average daily intake for humans, based on allometric scaling between the two species^46^. The average daily saccharin intake in humans is difficult to estimate because saccharin content of many products is not disclosed on the label. Therefore, in some cases human consumption of saccharin may exceed the recommended daily intake value. In any event, the 2% saccharin exposure used here is consistent with that employed in a number of rodent experimental models^47–55^.

Despite the 2% saccharin exposure in our model, we did not find significant changes in bodyweight of F0 males in the SAC group compared to those in the WATER group. However, the bodyweight of the F0 male mice in the SAC + NIC group was significantly lower than that of the F0 mice in the WATER group. It is possible that reduced food consumption in the SAC + NIC group may have contributed to the bodyweight reduction. Since we did not measure food consumption in the present study whether the reduction in bodyweight in the SAC + NIC group may be due to reduced food consumption remains an unresolved issue.

The use of saccharin as a reinforcing stimulus in experimental animal models of hedonic responses is common because it is 200–700 times sweeter than sucrose and has no nutritional value. Saccharin’s reinforcing effects occur via activation of the gustatory pathways and dopamine signaling in reward centers of the brain^2,3^. Saccharin-induced changes in the reward pathways may be associated with increased drug seeking behavior, which is consistent with impulsive behavior in rats selectively bred for high saccharin intake^24,25^. Impulsivity and drug seeking both are associated with dopamine receptor signaling mechanisms^56–58^. The locomotor hyperactivity in F0 male SAC mice in the present study is consistent with changes in dopamine neurotransmission as well^11,26^. Thus, there is compelling evidence that long term exposure to saccharin may alter dopamine receptor signaling in the brain. Nicotine exposure of adolescents and adults also alters dopamine signaling and cognitive function including attention and impulsivity^17,37,59–62^. Thus, exposure to saccharin alone or nicotine alone is associated with behavioral phenotypes consistent with impaired dopamine neurotransmission. These observations raise the possibility that co-exposure to saccharin and nicotine may produce “additive” effects on cognitive phenotypes associated with dopamine receptor signaling mechanisms in the F0 and F1 generations.

However, a comparison of the phenotypes between the F0 and F1 generations revealed two interesting points. First, the phenotypes in the F1 mice produced by co-exposures to saccharin and nicotine did not appear to represent additive effects of each substance. For example, although neither the F1 male offspring derived from the FO SAC group nor from the nicotine only exposed F0 group in a previous study^26^ showed significant changes in working memory, the F1 male mice derived from the SAC + NIC group did. Thus, saccharin and nicotine co-exposures led to working memory deficits in the F1 offspring whereas exposure to either substance alone did not. Second, neither the F0 SAC nor the F0 SAC + NIC male mice showed locomotor hyperactivity or working memory deficit. However, both these phenotypes were present in the F1 offspring derived from one or both these F0 groups. Thus, saccharin and nicotine exposures produced “emergent” phenotypes in the F1 generation that were not present in the F0 generation. These observations illustrate the challenges associated with understanding heritable phenotypes produced by exposures to combinations of stimuli such as tobacco smoke, e-cigarette liquid or air pollution. Understanding the effects of each component of the combination in isolation may not reveal the full spectrum of effects of the combination.

The transgenerational phenotypes associated with paternal saccharin only exposure may be associated with changes in DNA methylation in the F0 spermatozoa, especially at promoter regions of the dopamine D1, D4 and D5 receptor genes. However, there was no direct correlation between changes in F0 spermatozoal total DNA methylation and F1 behavioral phenotypes because co-exposure to saccharin and nicotine did not produce changes in total DNA methylation in the F0 spermatozoa but produced behavioral phenotypes in the F1 generation. Therefore, establishment of epigenetic changes in the spermatozoa of the F0 mice as a mechanism for heritability of behavioral phenotypes in the F1 generation must await further research including data on additional epigenetic changes such as changes in expression of noncoding RNA.

The duration of spermatogenesis (time taken for spermatogonia to develop into spermatozoa) is approximately 35 days in mice^40,63^. Therefore, the 8-weeks of exposure to saccharin or saccharin plus nicotine would have been sufficient to expose spermatozoa throughout the entire spermatogenesis cycle. However, the 8-week exposure did not produce transgenerational phenotypes—a 12-week exposure was necessary. Interestingly, in the case of paternal exposure to nicotine alone a 12-week exposure was necessary to produce heritable phenotypes^26^. It is possible that 12 weeks of exposure of spermatogonia as well as mature spermatozoa is required to produce heritable epigenetic changes in the spermatozoa. However, since spermatogonia are an asynchronously proliferating population, and phagocytosis or resorption of mature spermatozoa is an ongoing process, the minimum duration of exposure needed to produce heritable epigenetic changes is difficult to calculate accurately in our model.

The implications of saccharin’s behavioral effects and their transgenerational transmission reported here may be significant because saccharin is used extensively in food, drink and pharmaceutical products, since its reintroduction as a “safe” sweetener in the year 2000. Although the bitter aftertaste of saccharin is a drawback, and there are other sweeteners that do not have a bitter aftertaste, saccharin use prevails because of its heat stability and relatively low chemical reactivity. These characteristics contribute to longer shelf life of saccharin-containing products^64^. As a result, saccharin is the sweetener of choice in baked goods, in food products that are heated prior to consumption, and packaged foods. Saccharin is also mixed with less stable sweeteners such as aspartame in nearly all fountain diet drinks.

Co-exposure to saccharin and nicotine such as that modeled here occurs in users of smokeless tobacco products some of which contain 25-fold higher saccharin levels than those found in food products^8^. It is estimated that 3 in 100 individuals consume smokeless tobacco products in the United States^5^. Therefore, our finding that exposure to saccharin alone or co-exposure to saccharin and nicotine produce heritable adverse phenotypes, some of which are consistent with the symptoms of neurodevelopmental disorders such as attention deficit hyperactivity disorder should raise significant public health concerns. The effects of co-exposure are not merely additive, nor can they be predicted based on knowledge of the effects of exposure to each substance alone. These observations raise concerns about de novo phenotypes that could emerge in future generations as a result of exposures to a combination of multiple harmful environmental factors today.

## Methods

### Animals

Eight-week old-male C57BL/6 mice were purchased from Charles River Laboratories (Kingston, NY, USA) and pair-housed in our institutional Laboratory Animal Resources facility in a temperature and humidity-controlled environment on a 12-h light–dark cycle with food and water available ad libitum. One week after arrival, the mice were randomly assigned to one of three drinking water exposure groups: plain drinking water (WATER), drinking water containing 2% saccharin (SAC; Sigma, Cat# S1002) or drinking water 2% saccharin plus 200 µg/ml nicotine (SAC + NIC; Sigma, Cat# N3876). Water consumption and body weight were recorded weekly or every other week, respectively. The drinking water exposures continued for a total of 12 weeks. The WATER group of mice were part of an earlier study^26^, because all F0 mice for the present and earlier study were produced simultaneously.

Following 8- and 12-weeks of such exposures, and while the exposures were ongoing the male mice were bred with drug naïve female mice to produce the F1 generation of offspring. Litters were standardized to contain 6–8 offspring on postnatal day 0 (P0; day of birth). Upon weaning on P21, same-sex offspring were group housed. No more than 2–3 male and female mice each from a given litter were used in each of the following analyses. We refer to the male mice in the three drinking water exposure groups as F0 (founder) mice. Experimental procedures were approved by the Animal Care and Use Committee of the Florida State University and were in full compliance with the NIH Guide for the Care and Use of Laboratory Animals as well.

### Behavioral analyses

The following sequence was used: Spontaneous locomotor activity, spatial working memory, object-based attention and motor-impulsivity. For the F0 mice, the analyses began one week following the breeding, and for the F1 mice around P60. Immediately prior to the commencement of the behavioral analyses, mice were habituated to the testing room for at least 30 min. In all the behavioral tests, except the spontaneous locomotor activity test (see below), habituation and behavioral testing occurred during the lights-off period, when mice are naturally more active. Dim red light was used for ambient illumination and for video recording. In all cases the mice from the three paternal treatment groups were tested concurrently.

### Spontaneous locomotor activity

We used testing chambers equipped with motion sensors (Photobeam Activity System, San Diego Instruments, San Diego, CA, USA) which create a 3-dimensional grid (5.4 cm spacing) of infra-red beams within the entire chamber^26^. On the day of analysis mice were removed from their home cage and placed individually into testing chambers. As the mouse moved along the X or Y axes the number of breaks in the infrared beams were recorded at 60-min intervals over a 12-h period (19:00 to 07:00 h; daily lights off period). Each beam break represented an ambulatory event. Activity during the initial 2-h period upon introduction of the mouse into the test chamber, which represents activity during habituation to the novel testing environment, was not included in 12-h period of analysis of spontaneous locomotor activity^11,13^.

### Spatial working memory

A custom-built clear Plexiglas Y-maze was used^10,12,26^. The maze consisted of three arms (each arm was 35 cm long × 6 cm wide × 10 cm high) radiating from the center and organized in the shape of the letter Y. Each arm was randomly assigned a letter code (A, B, and C). Unique visual cues were placed on the walls of each arm as well as on the walls of the testing room to facilitate visual discrimination among the 3 arms by the mouse. The mouse was placed at the end of one of the arms and its activity recorded using an overhead video camera over a 6-min period as it explored the maze. The video recordings were analyzed by an investigator blinded to the identity of the mouse to calculate the number of entries into each arm (when all four limbs of the mouse enter an arm) and the sequence of arm entries. An “alternation” is a set of three consecutive non-repetitive arm choices (e.g. ABC, BCA, CBA). A percent spontaneous alternation score is # alternations ÷ (# of entries − 2) × 100.

### Motor-impulsivity

The cliff avoidance reaction (CAR) test was used to assay motor-impulsivity. The apparatus consisted of a custom-built circular Plexiglass platform (20 cm in diameter) supported on a plastic rod (50 cm in height) resembling to a barstool^12^. The mice were placed individually at the center of the platform and their behavior was recorded over a 60-min period using an overhead video camera. If a mouse fell off the platform, it was gently picked up and returned to the center of the platform. The video recordings were analyzed by an investigator blinded to the identity of the mouse to calculate the number of times each mouse fell off the platform. A CAR index was calculated for each experimental group and expressed as a percentage using the formula: (number of mice that fell off the platform ÷ total number of mice in the group) × 100.

### Attention

An object-based attention (OBA) test was used. The apparatus consisted of a custom-built rectangular, opaque Plexiglas box with a larger (training) chamber (40 cm × 40 cm × 25 cm) and a smaller (test) chamber (40 cm × 20 cm × 25 cm) separated by a sliding Plexiglas sliding divider wall. The assay consisted of 2 days of habituation, followed by a testing session on day 3^10,12,26^. On day 1 (habituation), the mouse was habituated to the empty apparatus for a total of 10 min (5 min in each chamber). On day 2, (habituation), the mouse was habituated to 5 objects, each made of the same wooden material, of the same size but different shapes, in the training chamber for 5 min. Next, on the same day, the mouse explored two of these objects selected randomly, in the test chamber for 5 min. Day 3 began with an additional shorter habituation period (3 min in each chamber), followed immediately by exploration of the 5 objects used on day 2 in the training chamber for 3 min. Following a 10 s interval, the door separating the chambers was slid open, and the mouse entered the test chamber to explore two objects one of which was a familiar object that was selected randomly from the 5 objects used in the training chamber, whereas the second object was a “novel” object to which the mouse had never been exposed. The familiar object was placed along the wall of the test chamber in a position analogous to its original position in the training chamber, while the novel object was placed near the wall opposite. The session lasted for 3 min. The behavior of the mouse was recorded with an overhead video camera. An investigator blinded to the identity of the subjects analyzed the video recordings to calculate the length of time spent with each of the two objects (novel and familiar). A recognition index was calculated and expressed as percentage using the formula: TN/(TF + TN) × 100, where TF and TN represent time spent during the test session exploring the familiar and the novel objects, respectively. We included in the analysis only those mice that spent at least 20 s with both objects in the test chamber, to minimize variability in the data.

### Administration of methylphenidate

Methylphenidate HCl (Sigma; M2892) was dissolved in 0.9% Sodium Chloride Injection USP, (sterile saline) and administered to the mice via oral gavage (0.75 mg/kg^43^) 15 min prior to the commencement of spontaneous locomotor activity test. Mice in the vehicle group received comparable volume of saline .

### Collection of spermatozoa

Following completion of behavioral testing mice were anesthetized using a mixture of Ketamine and Xylazine (100 mg/kg and 20 mg/kg; intraperitoneal) and decapitated. The testis and epididymis were removed, and mature spermatozoa were collected from the cauda epididymis using a double swim assay^26^. Briefly, cauda epididymis was placed in a petri dish with phosphate buffered saline (pH 7.2) containing 1% bovine serum albumin. Longitudinal cuts were made along the cauda epididymis to release the spermatozoa into the warm saline solution. The spermatozoa were incubated in saline at 37 °C for 30 min, to facilitate the sperm to “swim” into the supernatant. The supernatant was collected, and the sperm were given 10 min to “swim” into the supernatant again (“double swim assay”). A drop of the supernatant was smeared on a microscope slide and examined in a light microscope (Axiovert 25, Zeiss, Carl Zeiss, Thornwood, NY, USA) to examine sperm morphology and verify presence of mature spermatozoa. The remaining supernatant was centrifuged to produce a pellet which was frozen in liquid nitrogen and stored at − 80 °C until further analysis.

### DNA methylation and methylated DNA immunoprecipitation (MeDIP)

Genomic DNA was isolated from pelleted sperm using a ZR Genomic DNA –Tissue Microprep kit (Zymo ZRGenomic, Zymo Research, Irvine CA; Cat#: D3041), and quantified using a Qubit 2.0 fluorometer (Invitrogen, Carlsbad, CA, USA). Normalized genomic DNA was amplified using a Qiagen REPLI-g Whole Genome Amplification kit (Qiagen, Valencia, CA, USA; Cat#: 150023) according to the manufacturers’ protocol and prepared for anti-5-methylcytosine immunoprecipitation as follows; DNA (2 µg) was subjected to fragmented using a Diagenode Bioruptor 300 (30 s on; 90 s off; 7 cycles) to generate 200–500 bp fragments of genomic DNA. 1 µg of the disrupted DNA was stored as “input” DNA for later analysis. The remaining 1 µg of disrupted DNA was immunoprecipitated according to manufacturers’ protocol (Methylated-DNA IP Kit, Zymo Research, Irvine CA; Cat#: D5101). The recovered DNA was quantified using a Qubit 2.0 fluorometer. Random priming amplification of immunoprecipitated DNA was carried out using an Affymetrix 2.0 DNA polymerase (Thermo Fisher Scientific; Cat#: 70775X1000UN) and quantified using a Qubit 2.0 fluorometer.

Real-time thermal cycling was performed on input and immune precipitated DNA (StepOne Plus Thermocycler; Life technologies). All PCR reactions were performed in triplicate. Target DNA sequence quantities were estimated from threshold amplification cycle numbers (Tc). For every gene sequence studied, a ∆Tc value was calculated for each sample by subtracting the Tc value for the input DNA from the Tc value for the corresponding immunoprecipitated sample. DNA quantities were expressed as percentages of corresponding input using the following equation: % methylation = 2^−(∆Tc)^ × 100. Dopamine receptor primers were designed using NCBI and Methylprimer software as previously described (McCarthy et al., 2018).

### Statistical analysis

The main effects of treatment on water intake, body weight and behavioral parameters in F0 mice were analyzed using one-way analysis of variance (ANOVA). For behavioral analyses, the main effects of treatment (paternal drinking water exposure) and sex and treatment × sex interaction were analyzed using a two-way ANOVA. The spontaneous locomotor activity data were analyzed using a two-way repeated measures ANOVA or a mixed-effects model with treatment and time as the main factors. Post-hoc pair wise comparisons were performed using Bonferroni multiple comparisons test. A one-sample *t*-test was used to analyze the number of falls in the CAR. A two-tailed Student’s *t*-test was used to analyze the DNA methylation data, as differences between only two groups were evaluated. GraphPad Prism 8.2.1 software was used for all the statistical analyses. The number of mice in each experimental group for each study is shown in the figure legend.

## Data Availability

Upon request, all materials, data and associated protocols will be made available to readers promptly without undue qualifications in material transfer agreements.
